# Supplementary material for: Effectiveness of Online Mindfulness-Based Intervention (iMBI) on Inattention, Hyperactivity–Impulsivity, and Executive Functioning in College Emerging Adults with Attention-Deficit/Hyperactivity Disorder: A Study Protocol
Source: Int J Environ Res Public Health. 2021 Jan 30;18(3):1257. doi: 10.3390/ijerph18031257 (PMC7908460; doi:10.3390/ijerph18031257)
Supplement: Supplementary file 1 [file ijerph-18-01257-s001.pdf]

Table S1. The Proposed Online Mindfulness-Based Intervention Program.

Session Theme	Online Lecture Topics	In-Session Online Mindfulness Exercises	Offline Assignments	Areas of Intervention (Outcome Variables)
1. Introduction to ADHD and Mindfulness	• About the program<br>• Psychoeducation on ADHD<br>• Defining mindfulness<br>• Discussion of individual experiences with ADHD<br>• How purposefully changing one's awareness can change the quality of their experience	• Mindful Eating<br>• Mindfulness of Breath	• Formal: Daily 1-min mindfulness of breath exercise<br>• Informal: Mindful awareness of a routine daily activity (e.g., eating or brushing teeth)<br>• Informal: Mobile phone breathing exercise (taking a breath every time the phone rings) to improve awareness of breath in daily life	IA + HI + EF
2. ADHD Through the Lens of Mindfulness	• Offline practice review: Challenges in practicing formal practices and its relationship with ADHD symptoms (i.e. inattention and restlessness)<br>• Discussion of the acceptance-change dialectic<br>• Observing difficulties that arise during mindful breathing in session<br>• Discussion of helpful modifications to the formal practice such as counting, imagery, and walking	• Mindful Breathing<br>• Mindful Walking<br>• Brief Loving-Kindness	• Formal: Daily 3-min mindful breathing/mindful walking<br>• Informal: Mindful awareness of ADHD symptoms with curiosity (e.g., "What's my ADHD like?)"	IA + HI
3. Thinking, Noting, and Feeling	• Differences between thinking and noting and their relationship with feelings.<br>• Using the breath (or other sensory systems) as an "anchor" to bring awareness back into the present moment.<br>• Attentional check-in	• Mindful S.T.O.P.<br>• Mindfulness of Music and Sound<br>• Mindfulness of Movement<br>• Brief Loving-Kindness	• Formal: Daily 5-min mindfulness of breath, body, and sound exercise<br>• Informal: Mindfulness of daily life with an emphasis on sound and walking<br>• Informal: Practice attention check-ins (e.g., "Where is my attention right now?")	IA + HI

4. Mindful Awareness of Physical Sensation	• Being fully present with one's body<br>• Body movement and ADHD related symptoms (e.g., physical restlessness, body tension in response to stress)<br>• 'Auto pilot mode' versus 'mindful mode' in daily activities	• Mindful S.T.O.P.<br>• Body Scan<br>• Mindful Put-on-Shoes<br>• Brief Loving-Kindness	• Formal: Daily 5-min mindfulness of breath, body movement, sound, or body scan<br>• Informal: Mindful put-on-shoes<br>• Informal: Mindful placement of frequently lost items	IA + HI
5. Mindfully Attend to Inattention	• Mind like a lake and thoughts like boats metaphor<br>• Noticing inattention with openness and awareness<br>• ADHD and thoughts: Working with maladaptive thoughts	• Mindful S.T.O.P.<br>• Mindfulness of Thoughts<br>• Mindful Presence<br>• Brief Loving-Kindness	• Formal: Daily 8-min mindfulness of thoughts<br>• Informal: Counting the frequency of maladaptive thoughts in one day	IA + EF
6. Time Management with Mindfulness	• Psychoeducation on time management issues in ADHD<br>• Effective time management strategies with mindfulness	• Mindful S.T.O.P.<br>• Mindful Presence<br>• 5-min Time Management Toolkit<br>• Brief Loving-Kindness	• Formal: Daily 10-min mindful presence and 5-min time management toolkit<br>• Informal: Practicing loving-kindness with self and others	HI + EF
7. Mindful Awareness of Communication	• Interpersonal challenges in ADHD<br>• Introduction to mindful interaction<br>• S.T.O.P. as you talk	• Mindful S.T.O.P.<br>• Mindful Messaging<br>• Brief Loving-Kindness	• Formal: Daily 10-min mindful presence<br>• Informal: Mindful speaking and listening with a family member or friend	HI + EF
8. Review and Wrap-Up	• Making mindfulness part of daily life and how it takes practice to establish new mindfulness practice routines<br>• Ways to keep practicing (e.g., revision of iMBI program, smartphone applications)<br>• ADHD and mindfulness resources	• Mindful S.T.O.P.<br>• Mindful Presence<br>• Brief Loving-Kindness	• Designing personal plan of mindfulness practices based on the S.M.A.R.T. principle	EF

ADHD = Attention-Deficit/Hyperactivity Disorder; iMBI = Online Mindfulness-Based Intervention; IA = Inattention; HI = Hyperactivity–Impulsivity; EF = Executive Functioning; S.T.O.P. = Stop, Take, Observe, Proceed; S.M.A.R.T = Specific, Measurable, Attainable, Realistic, and Timely.
